# Seroprevalence of dengue IgG antibodies in symptomatic and asymptomatic individuals three years after an outbreak in Zhejiang Province, China

**DOI:** 10.1186/s12879-018-3000-5

**Published:** 2018-02-23

**Authors:** Shuying Luo, Weihong Cui, Chan Li, Feng Ling, Tao Fu, Qiyong Liu, Jiangping Ren, Jimin Sun

**Affiliations:** 1 Zhejiang Provincial Center for Disease Control and Prevention, Hangzhou, China; 2 Yiwu Municipal Center for Disease Control and Prevention, Yiwu, China; 3 Yantai Municipal Center for Disease Control and Prevention, Yantai, China; 4 State Key Laboratory for Infectious Disease Prevention and Control, Collaborative Innovation Center for Diagnosis and Treatment of Infectious Diseases, National Institute for Communicable Disease Control and Prevention, Chinese Center for Disease Control and Prevention, Beijing, China

**Keywords:** Dengue, Seroprevalence, Antibody, Duration

## Abstract

**Background:**

Cross-reacting antibodies enhanced dengue infection in humans and antibody dependent enhancement (ADE) have been proposed as early mechanisms underlying DHF/DSS. However, the duration of dengue IgG antibodies in the body as well as factors associated with said duration remain unclear.

**Methods:**

Blood samples from 59 dengue symptomatic persons and 48 asymptomatic individuals were collected. Study participant demographic information (including age in 2009, gender, and place of residence) were also collected. Serum samples were tested for dengue specific IgG by Panbio dengue IgG indirect enzyme-linked immunosorbent assay (ELISA). Chi-square tests and logistic regression analysis of dengue IgG antibodies seroprevalence divided by gender, age groups, and symptomatic or asymptomatic infection were conducted using the Statistical Package for the Social Sciences.

**Results:**

Overall, 70 (65.42%) blood samples were seropositive for dengue IgG antibodies with similar seroprevalences found when dividing by gender and different age groups. However, seroprevalence of dengue IgG antibodies in samples from dengue symptomatic persons was significantly higher than that in samples from asymptomatic individuals (96.61% vs 27.08%) according to multivariable logistic regression analysis, the odds ratio (OR) of the factor was 76.731.

**Conclusions:**

Dengue IgG antibodies were detectable in samples from most individuals three years after infection. Dengue symptomatic persons had a higher dengue IgG prevalence compared to asymptomatic individuals.

## Background

Dengue is one of the most prevalent mosquito-borne viral disease in humans and is caused by four distinct serotypes (DENV 1–4). DENV are mainly transmitted by *Aedes* mosquitoes and distributed in more than 100 countries in tropical and subtropical areas. More than 2.5 billion people are at risk of dengue infection in the world. The WHO estimates that more than 50 million dengue infections and 20,000 dengue-related deaths occur annually worldwide [1]. Another study estimated that there were 390 million dengue infections including 96 million apparent dengue infections in 2010 [2].

DENV cause a spectrum of diseases ranging from subclinical manifestations or a mild, self-limiting disease, dengue fever (DF), to a more severe disease, dengue hemorrhagic fever (DHF), which can progress to dengue shock syndrome (DSS) and death. Previous studies reported that cross-reacting antibodies enhanced dengue infection in humans and antibody dependent enhancement (ADE) had been proposed as the early mechanism underlying DHF/DSS [3–7]. Moreover, recent studies have reported that human antibody responses after dengue virus infection were highly cross-reactive with Zika virus and was able to drive ADE of Zika infection [8, 9]. Seroprevalence of dengue IgG antibodies was investigated in many countries where DENV are endemic. Jeewandara C et al. reported that 68.2% of individuals were seropositive for dengue in Sri Lanka and a significant rise in the age stratified seroprevalence rates was observed [10]. Mazaba-Liwewe ML et al. reported the first seroprevalence of dengue specific IgG antibodies in Western and North-Western provinces of Zambia indicating that 4.1% of the participants tested positive for dengue IgG in these areas [11]. Moreover, seroprevalence of dengue was also investigated in India, Thailand, Gabonese, Kenya, Saudi Arabia, Singapore, Tanzania, Sudan and factors associated with it were also explored [12–19]. However, the duration of dengue IgG antibodies and factors associated with duration remain unclear in China. Here, we investigated seroprevalence of dengue IgG antibodies among symptomatic persons and asymptomatic individuals three years after infection and analyzed associated factors.

## Methods

### Samples collection

In 2009, an outbreak of DENV-3 subtype III occurred in Yiwu, a city locates in central Zhejiang Province, which is located in Southeastern China and a total of 196 cases were identified in this outbreak [20]. Dengue cases are classified as probable case, clinically diagnosed case or confirmed case. Probable cases are those diagnosed by local experienced physicians according to cases’ epidemiologic exposure and clinical manifestations; clinically diagnosed cases are probable cases with positive DENV-specific IgM antibodies in their serum samples; confirmed cases are clinically diagnosed cases for which any of the following laboratory results are reported by the local public health institutes: fourfold or greater increase in DENV-specific IgG antibody titer between paired samples, or positive DENV polymerase chain reaction (PCR) test, or positive virus isolation and identification [21]. After this outbreak we conducted an investigation of asymptomatic infection and 102 asymptomatic individuals were identified during the outbreak [22]. In that study, serum samples were collected from persons who didn’t have medical visit history from July to September, 2009 and lived in the six villages where dengue outbreak occurred if they agreed with us. A person with no symptoms and dengue IgM was detected in his serum specimen was defined as an asymptomatic individual. In 2012, we collected blood samples from 59 dengue symptomatic persons and 48 asymptomatic individuals who agreed to the informed consent. No dengue outbreak occurred from 2009 to 2012 in Zhejiang Province, and all symptomatic persons and asymptomatic individuals in our study hadn’t traveled to dengue endemic areas during these years. Moreover, only one Japanese Encephalitis case were reported from 2004 to 2012 and no other flaviviruses were endemic in these villages where our samples were collected. Demographic data information of subjects including age in 2009, gender, and place of residence were collected. All the sera were then centrifuged, decanted, and stored at − 80 °C until testing.

### Samples detection

Serum samples were tested for dengue specific IgG by Australian Panbio dengue IgG indirect enzyme-linked immunosorbent assay (ELISA). The kits were used according to the manufacturers instructions. Panbio units were calculated according to the manufacturers instructions and > 11 units was defined as a positive result, 9–11 units was defined as a equivocal result, and < 9 units was defined as a negative result.

### Data analysis

Chi-square test was used to compare the difference in gender distribution age group distribution, and symptomatic or asymptomatic infection distribution across different serological status. Logistic regression analysis were conducted to explore factors associated with seroprevalence and non-parametric test was used to analyze the association between age value and sero-positivity. Data analysis were conducted using the Statistical Package for the Social Sciences (spss v20; SPSS, Chicago, IL, USA). The dependent variable in the logistic regression was assigned as the serological status of subjects and the independent variables were gender, age group, and symptomatic or asymptomatic individual. The method of logistic regression used was forward-conditional. The stepwise probability was set to 0.05 for entry and 0.10 for removal. The classification cutoff was 0.5 and the maximum number of iterations was 20.

## Results

### Study participant demographics

Blood samples were collected from 107 individuals including 59 symptomatic persons and 48 individuals with asymptomatic infections. Among all participants, 42 were male and 65 were female; when dividing by age, 12 (11.22%) were in the age group of 1–20 years, 18 (16.82%) were 21–40 years, 31 (28.97%) were 41–60 years, and 46 (42.99%) were ≥61 years. The average age of all subjects was 52 years (range 3–86 years) old. Differences of gender distribution and age distribution between symptomatic persons and asymptomatic infections were not significant (2 = 1.279, *P* = 0.258; 2 = 3.954, *P* = 0.266).

### Dengue-specific IgG results

Overall, 70 (65.42%) blood samples were seropositive for dengue IgG antibodies. Seroprevalence of dengue IgG antibodies was found to be similar between males (73.81%, *n* = 42) and females (60.00%, *n* = 65; Table 1). Sero-positive rates of the four age groups were 66.67%, 66.67%, 61.29%, and 67.39%, respectively (Table 1). Differences in seroprevalence between different age groups were insignificant (2 = 0.333, *P* = 0.954). Age didn’t have significant association with sero-positivity (Z = − 0.265, *P* = 0.791). However, seroprevalence of dengue IgG antibodies in samples from dengue symptomatic persons was significantly higher than those from asymptomatic individuals (96.61% vs 27.08%, Table 1). According to the results of multivariable logistic regression analysis, the variable in the final equation was the participant type (symptomatic or asymptomatic) and the odds ratio (OR) of the variable was 76.731 (95% CI: 16.334–360.451). Wals and B of the equation were 30.236 and 4.340, respectively.

**Table 1:** Seroprevalence of dengue IgG antibodies in people of different characteristics

	Negative	Positive	Total	Chi-square	*P*
Gender				2.151	0.142
Male	11	31	42		
Female	26	39	65		
Age group				0.333	0.954
1-	4	8	12		
21-	6	12	18		
41-	12	19	31		
61-	15	31	46		
Participant type				56.556	0
Symptomatic	2	57	59		
Asymptomatic	35	13	48		

## Discussion

In several provinces around China, the number of dengue outbreaks as well as dengue cases have increased in recent years [23, 24]. With climate warming, even more dengue cases are projected to occur in some areas of China in future [25]. Additionally, it is likely that some individuals who have been infected with one serotype of dengue virus will contract another serotype. Previous studies reported that dengue antibodies were detectable more than 60 years after infection [26, 27]. Up to date, this is the first study investigating the duration of dengue antibodies in China. In this study, we collected serum samples from dengue symptomatic persons and asymptomatic infections three years after infection and analyzed factors associated with seroprevalence.

The overall positive rate of dengue IgG antibodies among all subjects was above 65% indicating that dengue antibodies can last a long time after infection. False positive results may occur due to endemicity of Japanese Encephalitis in Zhejiang Province, China. We searched all Japanese Encephalitis cases reported from 2004 to 2012 in villages where our samples were collected. Only one case was reported during this period in these villages and no serum sample was collected from him. We can’t rule out false positive, but false positive rate should be very low. Furthermore, dengue is not endemic and no dengue outbreak occurred from 1950 to 2012 except the outbreak in 2009 in the study area. We can concluded that all infecting serotypes were DENV-3 related to the outbreak in 2009 and all symptomatic persons and asymptomatic individuals were all primary infection. According to previous reports [3–9], the existence of dengue antibodies could enhance dengue infection and Zika infection. Accordingly, we suggest that more attention should be paid to dengue or zika cases who have been infected with dengue viruses, even they contracted dengue viruses many years ago.

Of interest, sex and age were insignificant predictors of seroprevalence of dengue IgG antibodies three year after infection. This may be because gender and age are unrelated to duration of dengue IgG antibodies or the study’s small sample size. More research should be conducted to explore the relation between age of individuals infected with dengue and duration of dengue IgG antibodies.

Importantly, dengue symptomatic group showed significantly higher prevalence of dengue IgG antibodies than asymptomatic individuals three years after infection. The sensitivity of the kits used to detect dengue IgG antibodies may have leaded to false negative results. We also cannot rule out other extraneous factors that may have contributed to our results. We can infer that infection outcome is correlated with viral load and that dengue symptomatic persons may have initially contracted a higher load of dengue viruses than asymptomatic individuals. Dengue symptomatic persons had a higher titer of dengue IgG antibodies than asymptomatic individuals when they were infected. Hence, dengue antibodies were undetected in some serum samples from asymptomatic individuals after three years of decline. Nevertheless, dengue symptomatic persons exhibit higher levels of dengue IgG antibodies than asymptomatic individuals three years after infection.

There are several limitations in our study. First, serum samples were only diluted 1:100 for detection according to manufacturer instructions; the positive rates of dengue IgG antibodies for different dilution titers were not investigated. Second, we did not measure titers of dengue IgG antibodies of dengue symptomatic persons and asymptomatic individuals when they were identified in 2009. We do not know how much the antibody titre decreases within the 3 years. Third, serum samples of symptomatic persons and asymptomatic individuals were only collected three years after infection. Therefore, we do not know the dynamics of dengue antibodies after infection. These limitations suggested that antibodies and antibody titres should be detected in every year to explore antibody the dynamics in such seroprevalence studies.

## Conclusions

Despite the limitations stated above, our study confirmed that dengue IgG antibodies were detectable in samples from most individuals three years after infection and that dengue symptomatic persons showed higher levels of dengue IgG antibodies than asymptomatic individuals. The dynamics of dengue antibodies after infection and the mechanisms in seroprevalence between symptomatic and asymptomatic individuals begs further research.

## Abbreviations

ADE: Antibody dependent enhancement
DENV: Dengue virus
DF: Dengue fever
DHF: Dengue hemorrhagic fever
DSS: Dengue shock syndrome
ELISA: Enzyme-linked immunosorbent assay
